# A New Method for Image Protection Using Periodic Haar Piecewise-Linear Transform and Watermarking Technique †

**DOI:** 10.3390/s22218106

**Published:** 2022-10-22

**Authors:** Andrzej Dziech, Piotr Bogacki, Jan Derkacz

**Affiliations:** Institute of Telecommunications, Faculty of Computer Science, Electronics and Telecommunications, AGH University of Science and Technology, Mickiewicza 30, 30-059 Kraków, Poland

**Keywords:** watermarking, image protection, PHL transform, data embedding, multimedia systems

## Abstract

The paper presents a novel data-embedding method based on the Periodic Haar Piecewise-Linear (PHL) transform. The theoretical background behind the PHL transform concept is introduced. The proposed watermarking method assumes embedding hidden information in the PHL transform domain using the luminance channel of the original image. The watermark is embedded by modifying the coefficients with relatively low values. The proposed method was verified based on the measurement of the visual quality of an image with a watermark with respect to the length of the embedded information. In addition, the bit error rate (BER) is also considered for different sizes of a watermark. Furthermore, a method for the detection of image manipulation is presented. The elaborated technique seems to be suitable for applications in digital signal and image processing where high imperceptibility and low BER are required, and information security is of high importance. In particular, this method can be applied in systems where the sensitive data is transmitted or stored and needs to be protected appropriately (e.g., in medical image processing).

## 1. Introduction

There is a large number of areas where the security of multimedia content is crucial for ensuring privacy and citizens’ rights in general. Digital watermarking is an efficient and versatile technical means for embedding secret information into multimedia objects, such as still images, videos, and audio files. An example of such secret, sensitive information can be medical data related to patients. Watermarking technology can assure protection of the digital content against unauthorized access, tampering, sensitive information disclosure, or copyright infringement. Methods based on watermarking may be also used for such applications as steganography and pseudonymization of private data. A graphic or audio file marked in this way can help locate websites or FTP servers where these files are unlawfully shared. As a result, a digital watermark now has high hopes for an effective fight against fraud.

The efficient watermark should be characterized by the following features: Impercep-tibility—the watermark should be imperceptible to the human eye, and the inserted information should not deteriorate the visual quality of an original image. Robustness—the watermark is detectable even after the original image transformation and is difficult to be removed. Consideration of local image properties—the watermark is inserted with varying intensity in different areas, depending on the characteristics of the area (e.g., brightness) Watermark decoding method—the watermark can be read based on the watermarked image only, without the need to verify against the original image.

Image watermarking can be performed in the spatial or transform domain. Spatial domain methods usually result in direct modifications of image data, such as color bands, and brightness. The common method for embedding a watermark in the spatial domain is the Least Significant Bit (LSB) method where the secret information is inserted into the original image by modifying or replacing the least significant bits of pixels. On the other hand, transform-based techniques rely on changing spectral factors in the domain of a specific transform. To retrieve the image with an embedded watermark one needs to perform the corresponding inverse transform operation. Watermarks embedded in the transform domains are typically more reliable in comparison with the watermarks inserted in the spatial domain [1,2].

The most widely used transforms used in digital watermarking include discrete cosine transform (DCT) [3,4,5,6], discrete wavelet transforms (DWT) [7,8] and discrete Fourier transform (DFT) [9,10]. Combination of different transform methods can be implemented, (e.g., DCT and DWT transform) [11,12,13]. Additionally, transform-based techniques can be used jointly with other methods, such as, (e.g., singular value decomposition (SVD) [14] or discrete fractional random transform (DFRNT)) [2]. There are also new approaches that apply novel types of transforms that are orthogonal and can be parameterized [15].

In [16], Yan et al. presented a data hiding scheme based on LSB modification in the Piecewise-Linear Haar transform for audio signals. Yang et al. in [17] proposed a reversible data hiding method dedicated to images using symmetrical histogram expansion also in the domain of this transform.

However, Periodic Haar Piecewise-Linear (PHL) transform is only mentioned in the literature with regard to image compression tasks [18].

For obvious reasons medical images are private to the patient and authorized medical personnel and should be protected from unauthorized viewers. One method to protect such images is using cryptography including traditional symmetric cryptosystems and biometrics [19,20,21]. Digital content, in particular this related to medical images, is more and more often protected by a combination of tools, such as encryption and watermarking. As defined in [22] encryption algorithms can be considered as an “a priori” protection mechanism since once data is decrypted, it is no longer protected. A complement to “a priori” mechanism is “a posteriori” protection, which can be provided by watermarking.

Apart from unauthorized access to sensitive content, another potential threat to medical multimedia content is possible manipulations. Existing, widely available, image editing software and image altering tools allow us to easily manipulate a digital image nowadays. Studies of various image manipulation detection techniques are available in the literature. Numerous image forgeries that can be performed on the image and different image manipulation detection and localization methods were presented in [23]. Image manipulation can also concern biomedical sciences where the use of images to depict laboratory results is widely disseminated. Results published in [24] have shown an alarming level of image manipulation in the published record. A dedicated tool was used to detect some of the most common misbehaviors, running tests on a random set of papers and the full publishing record of a journal.

Currently, image tampering detection can be also realized with the use of Convolutional Neural Networks [25]. Image protection and manipulation detection are extremely relevant in all applications where the sensitive data is transmitted from the imaging sensor to a remote destination where it is further processed and analyzed [26]. Such protection can be realized in aerial photography, area monitoring, and satellite imagery [27]. The same applies to medical applications of remote sensing where electromagnetic radiation is most commonly the sensing medium and the sensors of diagnostic devices, which are exterior to the body of a patient, can detect various features of human tissues in a noninvasive way [28].

The paper is organized as follows. The next section is dedicated to Periodic Haar Piecewise-Linear Transform. Section 3 introduces a new method for data embedding. Section 4 presents the potential application of the proposed algorithm for the detection of image manipulations. In Section 5 the experimental results are presented and the comparison between the proposed solution and the DCT approach is discussed. Finally, Section 6 contains the conclusions and future work.

## 2. Periodic Haar Piecewise-Linear PHL Transform

This section covers the most important theoretical aspects related to Periodic Haar Piecewise-Linear (PHL) transform. The thorough description and further information are presented in detail in [29]. The Haar functions are defined by the following formulas:

(1a) $$\begin{array}{cc} har(0,t) & =1\;\mathrm{for}\;t\in (-\infty ,\infty ),\;\mathrm{usually}\;T=1 \end{array}$$

(1b) har(i,t)=−2k−12for[i2k−1−1]≤t<[i+122k−1]−2k−12for[i+122k−1−1]≤t<[i+12k−1−1]−0otherwise

where $0<k<\log_{2}N$, $1\leq i\leq 2^{k}$.

In turn, the PHL functions can be calculated by performing the integration of these Haar functions. It can be realized by using the below formulas:

(2a) $$\begin{array}{cc} \; & \mathrm{PHL}(0,t)=1\;t\in (-\infty ,\infty ) \end{array}$$

(2b) $$\begin{array}{cc} \; & \mathrm{PHL}\left(1,t\right)=\left[\frac{2}{T}\int_{mT}^{t+mT}har\left(1,\tau \right)d\tau \right]+\frac{1}{2} \end{array}$$

(2c) $$\begin{array}{cc} \; & \mathrm{PHL}\left(i+1,t\right)=\frac{2^{k+1}}{T}\int_{mT}^{t+mT}har\left(i+1,\tau \right)d\tau \end{array}$$

where $i=1,2,...,N-2;k=1,2,...,\log_{2}N-1;m=0,1,2,...$;

*k*—index of group of PHL functions;

*m*—number of period.

**Figure 1:**
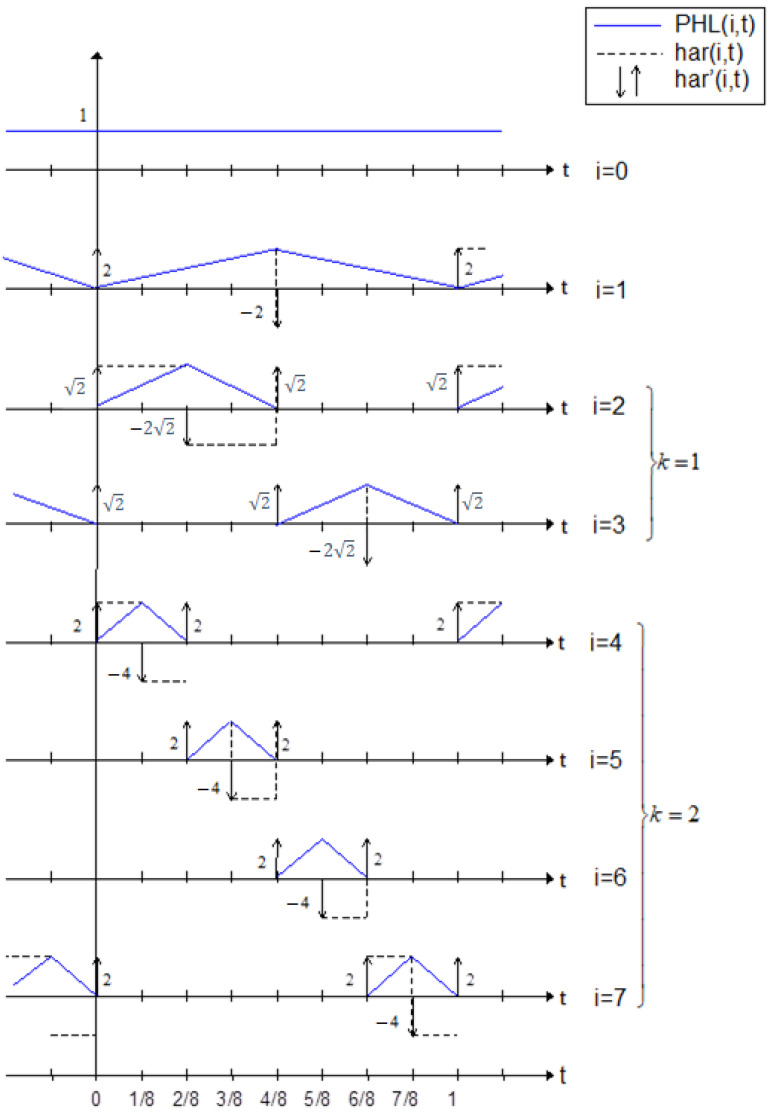
Set of PHL functions for N = 8.

Figure 1 depicts the derivatives (in distributive sense) of Haar functions. The PHL functions are linearly independent but they do not satisfy the orthogonality condition.

### 2.1. One-Dimensional PHL Transform

To perform forward and inverse PHL transform, the following matrix equations can be used:

a. Forward transform
  (3) $$\left[C(N)\right]=\left[-\frac{1}{2^{k+1}}\right]\left[\mathrm{PHL}(N)\right]\left[X(N)\right]$$
b. Inverse transform
  (4) [X(N)]=[IPHL(N)][C(N)]

where [C(N)]—vector of PHL coefficients (PHL spectrum);

[X(N)]—vector of sampled signal;

[PHL(N)]—matrix of forward transform;

[IPHL(N)]—matrix of inverse transform;

$\left[-\frac{1}{2^{k+1}}\right]$—diagonal matrix of normalization.

(5) $$\left[-\frac{1}{2^{k+1}}\right]=diag\left[1,-\frac{1}{2^{1}},-\frac{1}{2^{2}}\left(2\;times\right),-\frac{1}{2^{3}}\left(4\;times\right),...,,-\frac{1}{2^{k}}\left(2^{k-1}\;times\right)\right]$$

The first row of the forward transform matrix consists of number one at the first position and the remaining elements are equal to zero. Other rows are composed of derivatives (in a distributive sense) of periodic Haar functions. The matrix for the inverse transform [IPHL(N)] is constructed in such a way that particular rows consist of PHL function values calculated for the same argument. For instance, the [PHL(N)] and [IPHL(N)] matrices, for N = 8, are presented below:

(6) $$\left[\mathrm{PHL}(8)\right]=\left[\begin{array}{cccccccc} 1 & \;0 & \;0 & \;0 & \;0 & \;0 & \;0 & \;0\\ 2 & \;0 & \;0 & \;0 & \;-2 & \;0 & \;0 & \;0\\ \sqrt{2} & \;0 & \;-2\sqrt{2} & \;0 & \;\sqrt{2} & \;0 & \;0 & \;0\\ \sqrt{2} & \;0 & \;0 & \;0 & \;\sqrt{2} & \;0 & \;-2\sqrt{2} & \;0\\ 2 & \;-4 & \;2 & \;0 & \;0 & \;0 & \;0 & \;0\\ 0 & \;0 & \;2 & \;-4 & \;2 & \;0 & \;0 & \;0\\ 0 & \;0 & \;0 & \;0 & \;2 & \;-4 & \;2 & \;0\\ 2 & \;0 & \;0 & \;0 & \;0 & \;0 & \;2 & \;-4 \end{array}\right]$$

(7) $$\left[\mathrm{IPHL}(8)\right]=\left[\begin{array}{cccccccc} 1 & \;0 & \;0 & \;0 & \;0 & \;0 & \;0 & \;0\\ 1 & \;\frac{1}{4} & \;\frac{\sqrt{2}}{2} & \;0 & \;2 & \;0 & \;0 & \;0\\ 1 & \;\frac{1}{2} & \;\sqrt{2} & \;0 & \;0 & \;0 & \;0 & \;0\\ 1 & \;\frac{3}{4} & \;\frac{\sqrt{2}}{2} & \;0 & \;0 & \;2 & \;0 & \;0\\ 1 & \;1 & \;0 & \;0 & \;0 & \;0 & \;0 & \;0\\ 1 & \;\frac{3}{4} & \;0 & \;\frac{\sqrt{2}}{2} & \;0 & \;0 & \;2 & \;0\\ 1 & \;\frac{1}{2} & \;0 & \;\sqrt{2} & \;0 & \;0 & \;0 & \;0\\ 1 & \;\frac{1}{4} & \;0 & \;\frac{\sqrt{2}}{2} & \;0 & \;0 & \;0 & \;2 \end{array}\right]$$

In this case, according to Equation (5), the diagonal matrix of normalization takes the following form:

(8) $$\left[-\frac{1}{2^{k+1}}\right]=\left[\begin{array}{cccccccc} 1 & \;0 & \;0 & \;0 & \;0 & \;0 & \;0 & \;0\\ 0 & \;-\frac{1}{2} & \;0 & \;0 & \;0 & \;0 & \;0 & \;0\\ 0 & \;0 & \;-\frac{1}{4} & \;0 & \;0 & \;0 & \;0 & \;0\\ 0 & \;0 & \;0 & \;-\frac{1}{4} & \;0 & \;0 & \;0 & \;0\\ 0 & \;0 & \;0 & \;0 & \;-\frac{1}{8} & \;0 & \;0 & \;0\\ 0 & \;0 & \;0 & \;0 & \;0 & \;-\frac{1}{8} & \;0 & \;0\\ 0 & \;0 & \;0 & \;0 & \;0 & \;0 & \;-\frac{1}{8} & \;0\\ 0 & \;0 & \;0 & \;0 & \;0 & \;0 & \;0 & \;-\frac{1}{8} \end{array}\right]$$

It can be observed that:

(9) $$\left[-\frac{1}{2^{k+1}}\right]\left[\mathrm{PHL}(8)\right]\left[\mathrm{IPHL}(8)\right]=\left[I(N)\right]$$

where [I(N)] is the identity matrix.

### 2.2. Two-Dimensional PHL Functions and Transform

The 2D PHL transform can be formulated in the following way:

a. Forward transform
  (10) $$\left[C\left(N_{x},N_{y}\right)\right]=\left[-\frac{1}{2^{k_{y}+1}}\right]\left[\mathrm{PHL}\left(N_{y}\right)\right]\left[F\left(N_{x},N_{y}\right)\right]{\left[\mathrm{PHL}\left(N_{x}\right)\right]}^{T}{\left[-\frac{1}{2^{k_{x}+1}}\right]}^{T}$$
b. Inverse transform
  (11) $$\left[F\left(N_{x},N_{y}\right)\right]=\left[\mathrm{IPHL}\left(N_{y}\right)\right]\left[C\left(N_{x},N_{y}\right)\right]{\left[\mathrm{IPHL}\left(N_{x}\right)\right]}^{T}$$

where $\left[F\left(N_{x},N_{y}\right)\right]$—matrix of 2D signal;

$\left[C\left(N_{x},N_{y}\right)\right]$—matrix of coefficients (2D PHL spectrum);

$\left[\mathrm{PHL}\left(N_{y}\right)\right],\left[\mathrm{PHL}\left(N_{x}\right)\right]$—matrices of 1D PHL forward transform;

$\left[\mathrm{IPHL}\left(N_{y}\right)\right],\left[\mathrm{IPHL}\left(N_{x}\right)\right]$—matrices of 1D PHL inverse transform;

$\left[-\frac{1}{2^{k_{y}+1}}\right],\left[-\frac{1}{2^{k_{x}+1}}\right]$—diagonal matrices of normalization.

The non-periodic Haar Piecewise-Linear Transforms have an order (N+1) while the PHL Transforms have an order (*N*). Due to this fact, PHL transforms can be applied in digital signal and image processing since the data usually has a dimension that is a power of 2.

## 3. Data Embedding in PHL Spectrum

The watermarking approach, presented in this paper, is based on inserting secret information in the PHL transform domain. The method assumes that the PHL spectrum is calculated only for the luminance channel of the given image, representing its grayscale version. To speed up the computations, the forward transform operation is performed on smaller subimages, i.e., blocks with the size: 8 × 8 pixels, using Equation (10) and the matrices (6) and (7).

As a result, after performing the above process to the input signal, we get its spectral coefficients in the PHL domain. Typically, a limited number of these coefficients carry most of the signal energy [30,31].

**Figure 2:**
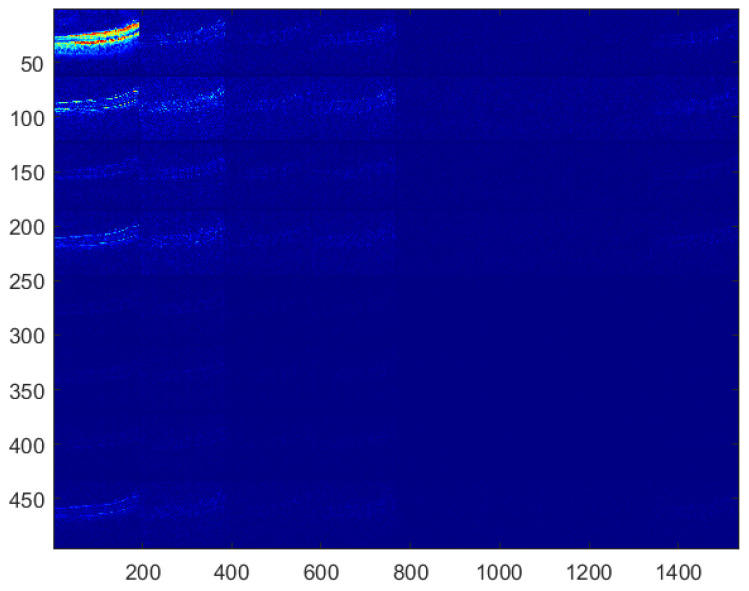
PHL spectrum coefficients grouped into channels.

The PHL transform may be used for image compression purposes [32]. In this task, the spectral coefficients that are above a given threshold are kept while the remaining ones are set to zero. Following this approach, our method assumes embedding of the watermark by modification of the coefficients having relatively low values. To perform this operation, the PHL coefficients are split into channels. Each channel groups the spectral coefficients with the same indices from each block processed in the forward transform step. This way, we obtain 64 PHL transform channels. The study of a set of various images and their spectra indicates that the top-left channel cumulates most of the signal energy. It is well depicted in Figure 2 which shows the PHL spectrum coefficients after grouping into 64 channels.

**Figure 3:**
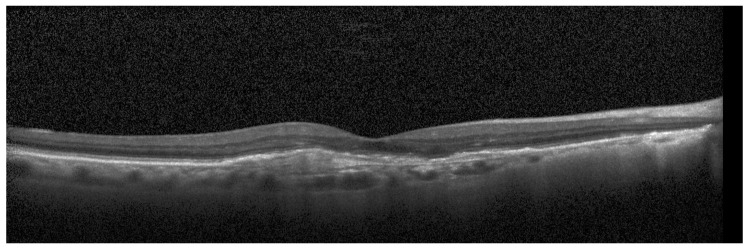
Sample OCT image.

**Figure 4:**
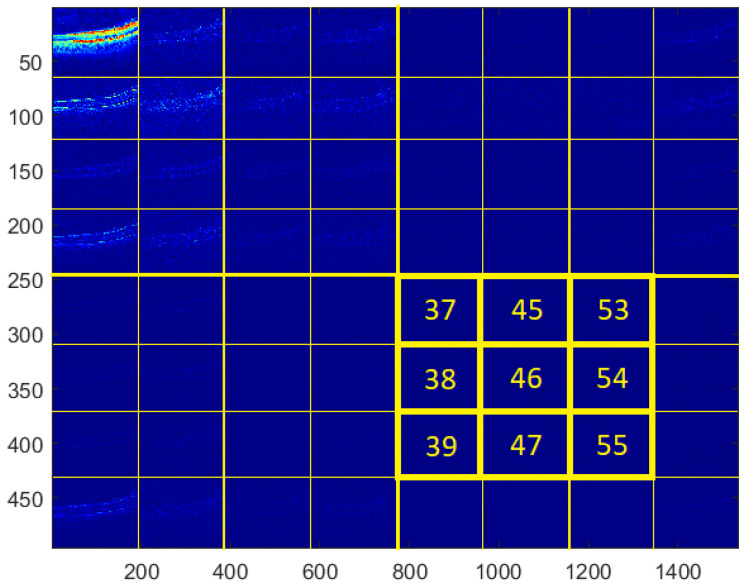
Blocks selected for data embedding.

For the testing purpose and the presentation of the image manipulation detection method in the following section, the Optical Coherence Tomography (OCT) images, having the resolution of 1536 × 496 pixels, were used [33]. The OCT is a non-invasive imaging examination that uses light waves to take cross-section pictures of the human retina. One sample image of this type is shown in Figure 3. The tests show that the blocks: 37–39, 45–47, and 53–55, marked in Figure 4, should be usually selected for the process of inserting secret information. This conclusion is based on the analysis of spectra of diverse images with varying content and characteristics. For the selection of the best channel for watermark embedding, the mean of all absolute values from each block is calculated. The channel with the lowest mean is chosen as the first candidate for the subsequent data embedding operation. To increase the capacity of the watermark, other blocks can be selected afterward, considering their mean values sorted in ascending order.

**Figure 5:**
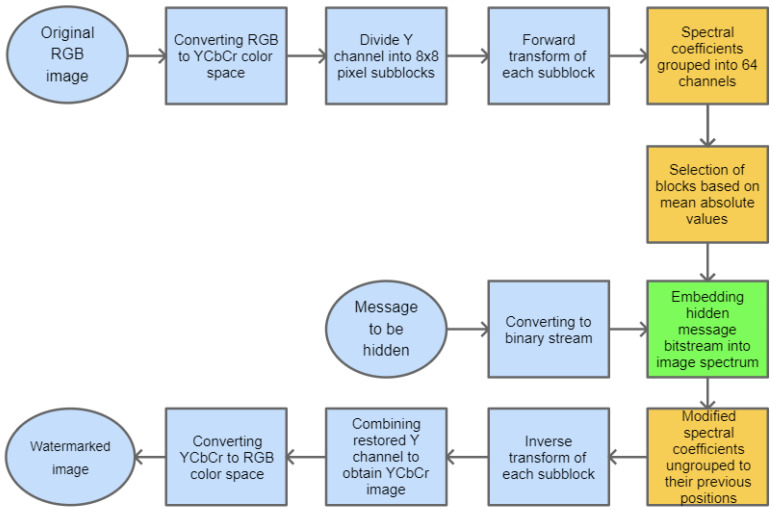
Block diagram for the base process of watermark embedding.

The selected channel coefficients are replaced with the consecutive bits of the message that is to be hidden in the image. Subsequently, the channel coefficients need to be relocated back to their previous positions. The final step is the inverse PHL transform of the modified image spectrum that results in the image with an inserted watermark. The stages of the whole embedding process are presented in Figure 5.

For the recovery of the embedded information, the same steps as previously need to be performed—the forward transform, the grouping of PHL coefficients, and finally extracting information from the selected channel or channels.

The selection of nine blocks for watermark embedding can be performed adaptively, as described previously, or arbitrarily. In this way, the chosen order can be used as an additional key at the watermark extraction phase.

## 4. Image Manipulation Detection

The information embedded as a watermark can be used to detect potential manipulations of the image. It would be beneficial if the hidden message could somehow describe the content of the image so that later, during the recovery phase, it could be compared with a newly generated description for the watermarked image. In case these two descriptions differ significantly, it could be stated that the watermarked image has been tampered with.

**Figure 6:**
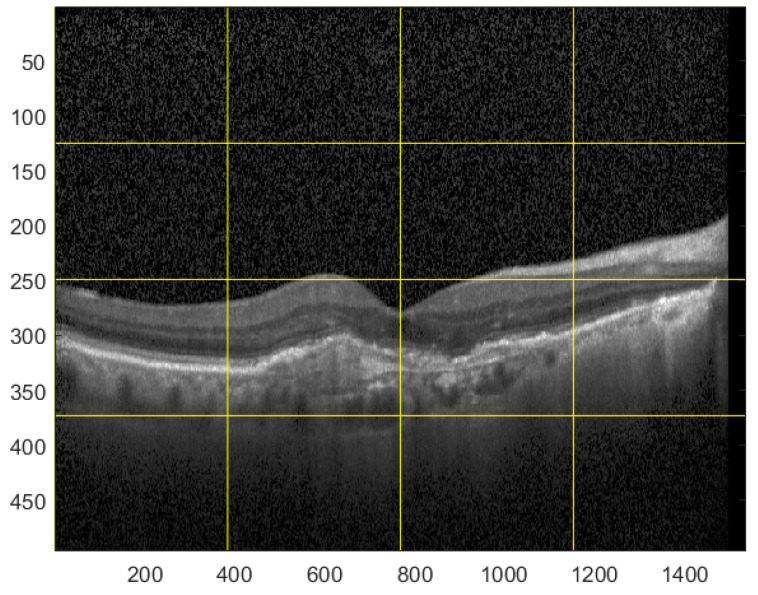
Blocks for which EHD descriptor is calculated.

In this paper, as a method for image description, MPEG-7 Edge Histogram descriptor (EHD) has been selected. It is a visual texture descriptor that captures the spatial distribution of five types of edges in an image: vertical, horizontal, two diagonals, and non-directional edge. It is created by dividing an input image into 16 (4 × 4) blocks, which is depicted in Figure 6. For each block, a histogram of all the above-mentioned types of edges is calculated. Therefore, it consists of 4×4×5=80 values that compose this descriptor [34].

In the first stage, the Edge Histogram descriptor is calculated for the given image. Its values are binarized to create a message bitstream which is then embedded into the image.

**Figure 7:**
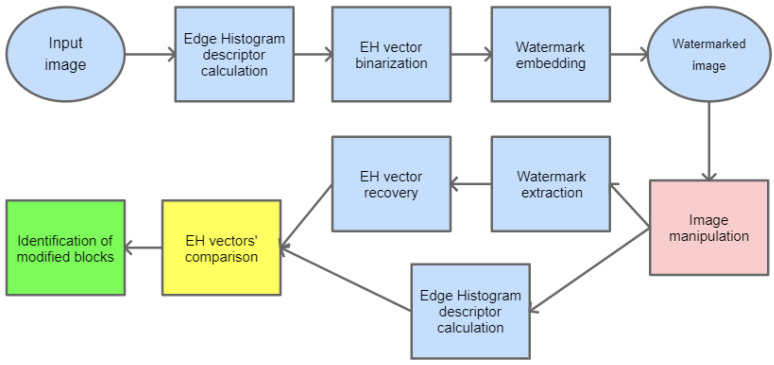
Block diagram for image manipulation detection process.

To detect potential manipulation of the watermarked image, it is necessary to calculate the EHD descriptor again and compare it with the one recovered from the watermark. The particular steps for image manipulation detection are shown in Figure 7.

**Figure 8:**
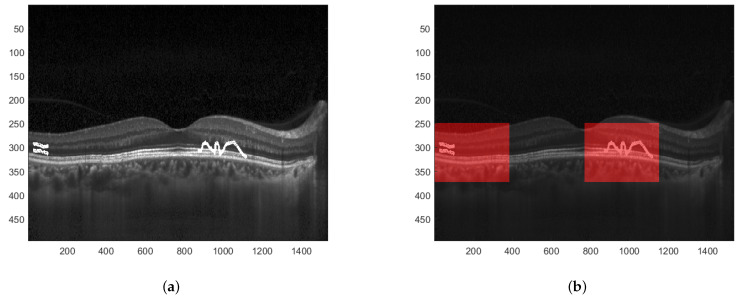
Result of image manipulation detection. (**a**) Tampered watermarked image. (**b**) Detected regions where the image has been manipulated.

When the difference between particular values of both descriptors is significant, one can determine that the image has been modified. Furthermore, since the EHD descriptor returns 5 values for each of the 16 blocks, the proper analysis of differences at the given positions can precisely indicate which of these 16 blocks have been tampered with. This is presented in Figure 8. A sample tampered image is presented in Figure 8a and the image with selected blocks that have been modified is shown in Figure 8b.

**Figure 9:**
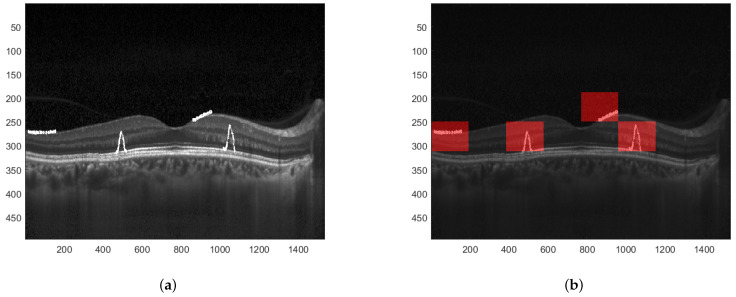
Result of image manipulation detection with greater precision. (**a**) Tampered watermarked image. (**b**) Detected regions with greater precision.

To obtain better precision for image manipulation detection the image can be initially divided into smaller sub-images which are then further processed following the same steps as in the previous example. In such a way, the blocks that are identified to have been tampered with are of smaller dimensions. This is depicted in Figure 9.

## 5. Experimental Results

The verification of the proposed algorithm is performed by measuring of Peak Signal to Noise Ratio (PSNR), which represents the visual quality of a watermarked image in relation to the total size of a watermark. Additionally, to consider the human visual system (HVS), Structural Similarity (SSIM) metric [35] and Universal Quality Image (UQI) index [36] are measured to assess the quality of the image with an embedded watermark. Furthermore, the bit error rate (BER) is also analyzed, for different lengths of the hidden message. The measurements of these ratios were performed for watermarks inserted in DCT and PHL transform domains so that the performance of both approaches may be compared. For test purposes, a random bit stream is used as a watermark message. The tests were carried out in a MATLAB environment. The referenced DCT method originates from the one described in [3].

For test purposes, 23 images from ‘Images 4k’ dataset [37] have been selected. The dataset contains 2057 files. The test images were selected in such a way that they represent different visual characteristics, i.e., low and high contrast and brightness as well as various color distributions. The dimensions of the images were reduced by half to 1920 × 1080 size so that the calculations and the watermark embedding process are speeded up.

**Figure 10:**
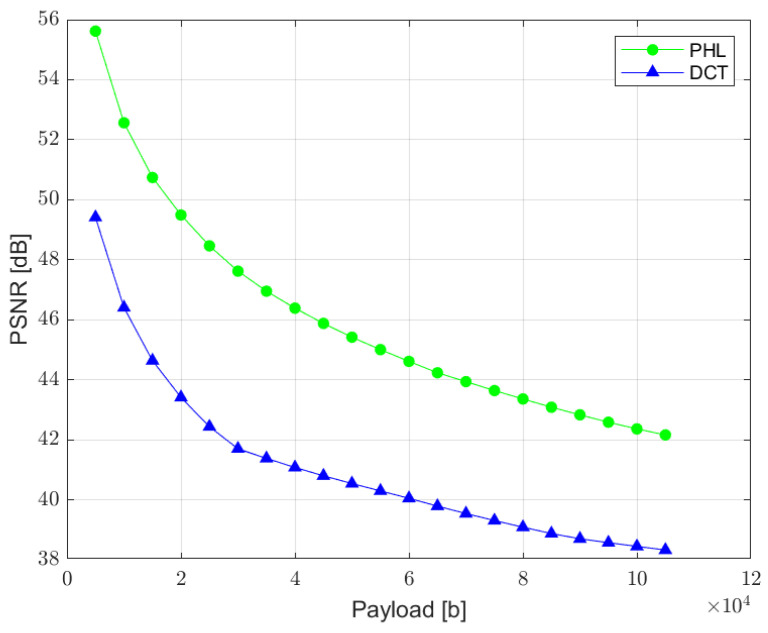
Relation between PSNR ratio and the watermark capacity (PHL vs. DCT).

The relation between the PSNR ratio and the length of a hidden bit stream is presented in Figure 10. It can be observed that a perceptual quality of an image with a watermark inserted in the PHL spectrum is consistently better than in the case of a watermark embedded in the DCT domain. It is assumed that the PSNR above 35 dB indicates that the two images being compared are visually identical, with no perceptual loss of quality [38]. Therefore, both techniques provide satisfying results as far as the imperceptibility of a watermark is concerned, for the size of a watermark exceeding even 100,000 bits.

**Figure 11:**
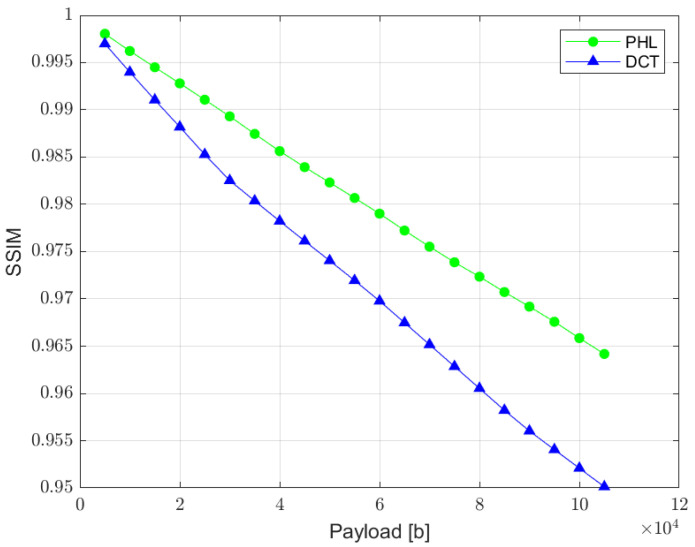
Relation between SSIM metric and the watermark capacity (PHL vs. DCT).

SSIM is a quality assessment metric based on the visual changes in local structure and contrast between two images. It provides a good approximation of human visual perception. The metric values can range from 0 to 1, where 1 indicates perfect similarity [35]. The relation between SSIM and the total size of a watermark is presented in Figure 11. The results measured for the PHL method are slightly better than the ones achieved in the DCT approach. However, both methods according to this metric provide satisfying results.

**Figure 12:**
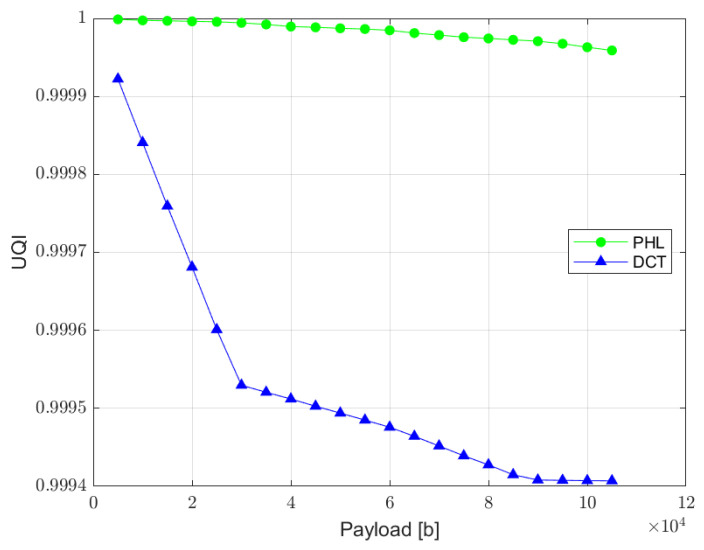
Relation between UQI index and the watermark capacity (PHL vs. DCT).

UQI index is designed to model image distortion as a combination of three factors: loss of correlation, luminance distortion, and contrast distortion. Although it does not employ any human visual system model, it was proved to be consistent with subjective quality assessment [36]. UQI index can vary between −1 and 1, where value 1 indicates no distortion present in the image. The relation between UQI and the length of a hidden message is presented in Figure 12.

**Figure 13:**
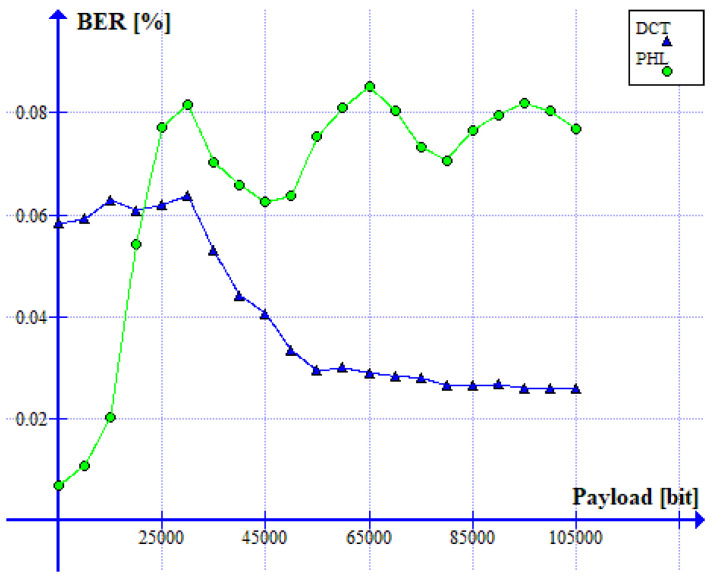
Relation between BER ratio and the watermark capacity (PHL vs. DCT).

The relation between the BER ratio and the size of a watermark is shown in Figure 13. It can be noticed that both methods guarantee a low bit error rate (<0.1%) for the watermark size ranging from 5000 to 105,000 bits. Therefore, both solutions are useful when a limited, but still, in most applications, sufficient, amount of information needs to be hidden in an image.

## 6. Conclusions and Future Work

We have presented a new watermarking scheme that is based on inserting a message bitstream in the PHL transform domain. The method offers a high capacity for hidden information and simultaneously satisfies the initial requirements of low image distortion and high accuracy during the watermark recovery stage. Therefore, it is a promising technique that can be used in a wide range of multimedia systems and services with emphasis put on medical applications where the aforementioned conditions need to be met. In addition, a method for the detection of image manipulation has been presented.

Further investigations will cover potential enhancements so that the method could be robust to various types of attacks. Finally, we plan to apply our solution in many applications in the upcoming future.

## Data Availability

Not applicable.

## References

[1] Sharma P.K., Sau P.C., Sharma D. Digital image watermarking: An approach by different transforms using level indicator. Proceedings of the 2015 Communication, Control and Intelligent Systems (CCIS).

[2] Zhou N.R., Hou W.M.X., Wen R.H., Zou W.P. (2018). Imperceptible digital watermarking scheme in multiple transform domains. Multimed Tools Appl..

[3] Lan T.-H., Tewfik A.H. (2006). A novel high-capacity data-embedding system. IEEE Trans. Image Process..

[4] Kim W.-H., Hou J.-U., Jang H.-U., Lee H.-K. (2018). Robust Template-Based Watermarking for DIBR 3D Images. Appl. Sci..

[5] Li H., Guo X. (2018). Embedding and Extracting Digital Watermark Based on DCT Algorithm. J. Comput. Commun..

[6] Xu Z.J., Wang Z.Z., Lu Q. (2011). Research on Image Watermarking Algorithm Based on DCT. Procedia Environ. Sci..

[7] Zhou X., Zhang H., Wang C. (2018). A Robust Image Watermarking Technique Based on DWT, APDCBT, and SVD. Symmetry.

[8] Narang M., Vashisth S. (2013). Digital Watermarking using Discrete Wavelet Transform. Int. J. Comput. Appl..

[9] Li L., Bai R., Lu J., Zhang S., Chang C.-C. (2021). A Watermarking Scheme for Color Image Using Quaternion Discrete Fourier Transform and Tensor Decomposition. Appl. Sci..

[10] Liao X., Li K., Yin J. (2017). Separable data hiding in encrypted image based on compressive sensing and discrete fourier transform. Multimed Tools Appl..

[11] Hasan N., Islam M.S., Chen W., Kabir M.A., Al-Ahmadi S. (2021). Encryption Based Image Watermarking Algorithm in 2DWT-DCT Domains. Sensors.

[12] Hazim N., Saeb Z., Hameed K. (2019). Digital Watermarking Based on DWT (Discrete Wavelet Transform) and DCT (Discrete Cosine Transform). Int. J. Eng. Technol..

[13] Akter A., Nur-E-Tajnina, Ullah M. Digital image watermarking based on DWT-DCT: Evaluate for a new embedding algorithm. Proceedings of the 2014 International Conference on Informatics, Electronics & Vision (ICIEV).

[14] He Y., Hu Y. A Proposed Digital Image Watermarking Based on DWT-DCT-SVD. Proceedings of the 2018 2nd IEEE Advanced Information Management, Communicates, Electronic and Automation Control Conference (IMCEC).

[15] Bogacki P., Dziech A. (2022). Analysis of New Orthogonal Transforms for Digital Watermarking. Sensors.

[16] Yan D., Wang R. Data Hiding for Audio Based on Piecewise Linear Haar Transform. Proceedings of the 2008 Congress on Image and Signal Processing.

[17] Yang L., Hao P., Zhang C. Progressive Reversible Data Hiding by Symmetrical Histogram Expansion with Piecewise-Linear Haar Transform. Proceedings of the 2007 IEEE International Conference on Acoustics, Speech and Signal Processing—ICASSP ’07.

[18] Dziech A., Tibken B., Slusarczyk P. Image compression using periodic Haar piecewise-linear PHL transform. Proceedings of the 2002 14th International Conference on Digital Signal Processing Proceedings.

[19] Abdallah H.A., ElKamchouchi D.H. (2022). Signing and Verifying Encrypted Medical Images Using Double Random Phase Encryption. Entropy.

[20] Lim E.Y.S. (2008). Data security and protection for medical images. Biomed. Inf. Technol..

[21] Fornazin M., Netto D.B., Cavenaghi M.A., Marana A.N. (2008). Protecting Medical Images with Biometric Information. Advances in Computer and Information Sciences and Engineering.

[22] Bouslimi D., Coatrieux G. (2015). Encryption and Watermarking for medical Image Protection. Medical Data Privacy Handbook.

[23] Thakur R., Rohilla R. (2020). Recent advances in digital image manipulation detection techniques: A brief review. Forensic Sci. Int..

[24] Bucci E.M. (2018). Automatic detection of image manipulations in the biomedical literature. Cell Death Dis..

[25] Wei X., Wu Y., Dong F., Zhang J., Sun S. (2019). Developing an Image Manipulation Detection Algorithm Based on Edge Detection and Faster R-CNN. Symmetry.

[26] Yuan G., Hao Q. (2020). Digital watermarking secure scheme for remote sensing image protection. China Commun..

[27] Zhu P., Jiang Z., Zhang J., Zhang Y.J., Wu P. (2021). Remote Sensing Image Watermarking Based on Motion Blur Degeneration and Restoration Model. Optik.

[28] Short N.M. Remote Sensing Tutorial: Medical Applications of Remote Sensing. https://drr.ikcest.org/remote-sensing-tutorial/introduction/Part2_26b.html.

[29] Dziech A., Bogacki P., Derkacz J. A Novel Watermark Method for Image Protection Based on Periodic Haar Piecewise-Linear Transform. Proceedings of the International Conference on Multimedia Communications, Services and Security, Communications in Computer and Information Science.

[30] Dziech A., Belgassem F., Nern H.J. (2000). Image Data Compression using Zonal Sampling and Piecewise-Linear Transforms. J. Intell. Robot. Syst..

[31] Baran R., Wiraszka D. (2015). Application of Piecewise-Linear Transforms in Threshold Compression of Contours. Logistyka.

[32] Dziech A., Ślusarczyk P., Tibken B.R. (2004). Methods of Image Compression by PHL Transform. J. Intell. Robot. Syst..

[33] Kermany D., Goldbaum M., Cai W. (2018). Identifying Medical Diagnoses and Treatable Diseases by Image-Based Deep Learning. Cell.

[34] Won C., Park D., Park S. (2002). Efficient Use of MPEG7 Edge Histogram Descriptor. Etri J..

[35] Wang Z., Bovik A., Sheikh H., Simoncelli E. (2004). Image Quality Assessment: From Error Visibility to Structural Similarity. IEEE Trans. Image Process..

[36] Wang Z., Bovik A.C. (2002). A universal image quality index. IEEE Signal Process. Lett..

[37] ‘Images 4k’ Dataset from Kaggle. https://www.kaggle.com/evgeniumakov/images4k.

[38] Aherrahrou N., Tairi H. (2015). PDE based scheme for multi-modal medical image watermarking. Biomed Eng. Online.

